# A Signal On-Off Ratiometric Molecularly Imprinted Electrochemical Sensor Based on MXene/PEI-MWCNTs Signal Amplification for the Detection of Diuron

**DOI:** 10.3390/bios15070433

**Published:** 2025-07-05

**Authors:** Yi He, Jin Zhu, Libo Li, Tianyan You, Xuegeng Chen

**Affiliations:** 1 Key Laboratory of Modern Agricultural Equipment and Technology, Ministry of Education, School of Agricultural Engineering, Jiangsu University, Zhenjiang 212013, China 2112116005@stmail.ujs.edu.cn (Y.H.); zhujin@ptl1.wecom.work (J.Z.); lbli@ujs.edu.cn (L.L.); 1000005204@ujs.edu.cn (X.C.); 2 College of Agricultural Equipment Engineering, Henan University of Science and Technology, Luoyang 471003, China

**Keywords:** ratiometric electrochemical sensor, molecularly imprinted polymer, MXene, Diuron, [Fe(CN)_6_]^3−/4−^

## Abstract

Diuron (DU) is a widely used phenylurea herbicide designed to inhibit weed growth, but its high toxicity and prolonged half-life contribute significantly to environmental contamination. The majority of electrochemical (EC) sensors typically rely on a single response signal for the detection of DU, rendering them highly susceptible to interference from variable background noise in complex environments, thereby reducing the selectivity and robustness. By integrating molecularly imprinted polymer (MIP) with a ratiometric strategy, the aforementioned issues could be solved. In this study, a novel signal on-off ratiometric MIP-EC sensor was developed based on the MXene/PEI-MWCNTs nanocomposite for the detection of DU. Positively charged PEI-MWCNTs was used as an interlayer spacer and embedded into negatively charged MXene by a simple electrostatic self-assembly method. This effectively prevented the agglomeration of MXene and enhanced its electrocatalytic performance. The MIP was synthesized via electropolymerization with DU serving as the template molecule and the selectivity was enhanced by leveraging the gate effect of MIP. Subsequently, a ratiometric MIP-EC sensor was designed by introducing [Fe(CN)_6_]^3−/4−^ into the electrolyte solution as an internal reference. Additionally, the current ratio signal (I_DU_/I_[Fe(CN)6]_^3−/4−^) and DU concentration exhibited a good linear relationship within the range of 0.1 to 100 µM, with a limit of detection (LOD) of 30 nM (S/N = 3). In comparison with conventional single-signal MIP-EC sensing, the developed ratiometric MIP-EC sensing demonstrates superior reproducibility and accuracy. At the same time, the proposed sensor was successfully applied to the quantitative analysis of DU residues in soil samples, yielding highly satisfactory results.

## 1. Introduction

As a type of phenylurea herbicide, Diuron (DU) effectively controls the growth of weeds by blocking plant photosynthesis, thereby enhancing crop yields [1]. However, due to its stable structure, DU is difficult to degrade naturally and becomes a persistent environmental pollutant [2]. It readily penetrates the soil to contaminate water bodies and poses a severe threat to aquatic organisms [3]. The extant research findings indicate that DU has the potential to exert carcinogenic effects on mammals [4]. It is evident that the high toxicity and long half-life of DU have led to its classification as a pollutant that requires prioritized control [5]. Consequently, the monitoring of DU in the environment is imperative for the preservation of human health. In recent years, electrochemical measurement has gained considerable popularity due to its low cost, rapid detection, and ease of operation [6,7]. As an inert electroactive substance, DU can generate an electrical signal at a high potential, thus prompting researchers to develop various highly sensitive EC sensors [8]. Nevertheless, enhancing the selectivity and accuracy of electrochemical detection remains a significant challenge.

The combination of molecular imprinting technology and the ratiometric strategy might constitute an effective approach to overcome these challenges. Molecularly imprinted polymer (MIP) is a class of intelligent materials featuring three-dimensional imprinted cavities that can precisely match the size, structure, and functional groups of template molecules [9,10]. Furthermore, an extra signal is introduced as an inherent correction factor, which is internally calibrated by calculating the ratio between the electrochemical response of the analyte to the internal reference signal [11]. This method has been shown to mitigate the unpredictable influences caused by individual or environmental variations. Liu et al. have developed a ratiometric MIP-EC sensor by integrating the MIP with in situ chemically reduced silver nanoparticles (AgNPs) on a carbon cloth (CC) modified with nitrogen-sulfur co-doped porous carbon (NSC) [12]. The latter serves as a reference signal for the precise detection of chlorpromazine. However, this method necessitates the immobilization of the reference probe onto the nanomaterial surface, which poses challenges in achieving high binding stability between the reference probe and the nanomaterials. Researchers have demonstrated that the direct incorporation of an internal reference into the detection solution effectively addresses the aforementioned challenges [13], offering a novel perspective for developing ratiometric molecularly imprinted electrochemical (MIP-EC) sensor.

In order to enhance the sensitivity of the MIP-EC sensor, it is necessary to implement signal amplification. Signal amplification in the MIP-EC sensor can be achieved through various strategies, such as optimizing the electrode material structure to increase the imprinted sites [14,15]. The combination of nanomaterials and MIP contributes to constructing high-performance electrochemical sensors [16,17]. Among the realm of nanomaterials, MXene is favored for its unique layered structure. This nanomaterial, which is classified as two-dimensional (2D) transition metal carbide or carbonitride, is favored for its high conductivity and outstanding mechanical strength [18]. Due to its unique layered structure, MXene holds promise as a support material, providing enhanced spatial capacity for the immobilization of functional nanomaterials [19]. However, MXene exhibits a strong tendency towards interlayer aggregation, which limits its application in EC sensors. To address this challenge, highly conductive multilayer carbon nanotubes (MWCNTs) are employed as interlayer spacers between MXene nanoplates. The introduction of MWCNTs not only inhibits the aggregation propensity of MXene, but also facilitates electron transfer and enhances the surface area. Typically, pristine MWCNTs bear a negative charge and exhibit poor dispersion [20]. Consequently, it is imperative to modify and combine MWCNTs with MXene to prepare the electrode modification material that exhibits exceptional electrochemical properties.

**Scheme 1:**
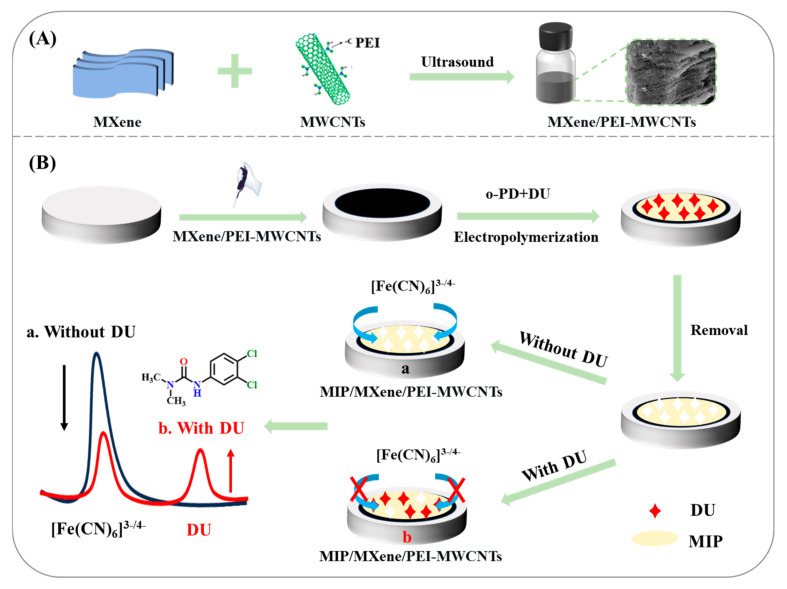
Schematic illustration of (**A**) the production of MXene/PEI-MWCNTs nanocomposite and (**B**) the preparation of ratiometric MIP-EC sensor.

Herein, a novel signal on/off ratiometric MIP-EC sensor was proposed for detecting DU. PEI-MWCNTs with positive charges were obtained by polyethylenimine (PEI) modification and their dispersion properties were improved. Subsequently, MXene/PEI-MWCNTs nanocomposite was prepared as substrate using electrostatic self-assembly to enhance the detection sensitivity. Furthermore, the MIP layer was synthesized through electropolymerization on the modified electrode surface, with o-phenylenediamine (o-PD) serving as the functional monomer and DU being utilized as the template molecule. Finally, a novel ratiometric MIP-EC sensor was fabricated by introducing [Fe(CN)_6_]^3−/4−^ as an internal reference in the electrolyte solution (Scheme 1). This strategy served to mitigate the impact of the electrode state and environmental variations on the detection outcomes. The quantification of DU was ascertained by calculating the ratio of the oxidation peak current of DU to [Fe(CN)_6_]^3−/4−^, expressed as I_DU_/I_[Fe(CN)6]_^3−/4−^, for the detection of DU in soil samples. Moreover, there are no reports on the application of ratiometric MIP-EC sensors to detect DU.

## 2. Experimental Section

### 2.1. Synthesis of MXene/PEI-MWCNTs

Initially, 40 mg MWCNTs and 400 mg PEI were dispersed in 40 mL ultrapure water, followed by sonication for 1 h to acquire the reaction mixture. Subsequently, the suspension was centrifuged three times with ultrapure water at 10,000 rpm to remove any impurities and then dried at 60 °C for 12 h, thereby obtaining PEI-MWCNTs powder. Furthermore, 20 mg PEI-MWCNTs and 10 mg MXene were dispersed in 10 mL ultrapure water, underwent sonicated for 30 min. Then, the suspension was centrifuged and washed three times with ultrapure water to remove the unwanted substances and adsorbed impurities, and the resulting MXene/PEI-MWCNTs nanocomposite was acquired after drying at 60 °C.

### 2.2. Construction of MIP/MXene/PEI-MWCNTs/GCE

The glassy carbon electrode (GCE) was polished with alumina slurry. After polishing, the electrode was cleaned by sonicating (at 40 kHz) with ultrapure water and ethanol to obtain a clean GCE. First, 6 mg MXene/PEI-MWCNTs nanocomposite was dispersed in 3 mL ultrapure water and sonicated for 1 h to disperse it evenly. Then, the 2.5 μL suspension was placed onto a clean GCE surface and the resulting MXene/PEI-MWCNTs/GCE was dried at room temperature.

MIP/MXene/PEI-MWCNTs/GCE was prepared by electropolymerization. First, the MIP/MXene/PEI-MWCNTs/GCE was dipped in 0.1 M acetate buffer solution (ABS), which contained 2 mM DU and 6 mM o-PD. The electropolymerization was carried out by cyclic voltammetry (CV) at a potential of −0.2 to 1.0 V for 15 cycles, with a scan rate of 50 mV/s. Then, the prepared electrode was soaked in an ethanol-acetic acid mixture (V_1_:V_2_ = 9:1) for 150 s to eliminate DU molecules. Eventually, the electrode was cleaned using ultrapure water and air-dried to yield the MIP/MXene/PEI-MWCNTs/GCE. Furthermore, for comparison, a non-molecularly imprinted electrochemical sensor (NIP/MXene/PEI-MWCNTs/GCE) was prepared without adding the DU molecules during the electropolymerization process and underwent the same cleaning process as the MIP one [17].

### 2.3. Electrochemical Measurements

The electrochemical experiment was based on the traditional three-electrode system, with the MIP/MXene/PEI-MWCNTs/GCE was employed as the working electrode, a platinum wire was utilized as the counter electrode, and an Ag/AgCl electrode was adopted as the reference electrode. The electrochemical impedance spectroscopy (EIS) measurements were performed using a voltage amplitude of 10 mV over frequencies ranging from 0.01 Hz to 100 kHz. The ZSimDemo (3.30d) software was used for the interpretation of the EIS spectra. Differential pulse voltammetry (DPV) was utilized to quantify DU within the voltage range of 0 to 1.3 V, employing a modulation amplitude of 50 mV and a pulse width of 0.02 s. First, the MIP/MXene/PEI-MWCNTs/GCE was submerged in the standard solution of DU for 10 min to combine the target analyte. Finally, the DPV measurement was performed in the electrolyte solution comprising 40 mM Britton–Robinson (BR, pH 6.5) buffer solution, 10 μM K_3_[Fe(CN)_6_], and 10 μM K_4_[Fe(CN)_6_].

### 2.4. Determination of Real Samples

The recovery of DU in soil samples was measured by a standard addition method to evaluate the practicality of the ratiometric MIP-EC sensor [21]. The soil samples were collected from cotton fields in Tumushuke, Xinjiang (China). First, the soil samples were sieved. Then, 5 g crushed soil sample was added to a mixture of 5 mL water and 10 mL acetonitrile, and the mixture was shaken for 15 min to facilitate extraction. Next, 5 g NaCl was added to promote phase separation. The mixture was subjected to centrifugation at a speed of 6000 r/min for 10 min. Eventually, the supernatant was filtered through a 0.22 μm microfiltration membrane. Various concentrations of DU were spiked into the treated samples, followed by sonication for 5 min to obtain spiked samples with different DU concentrations.

## 3. Results and Discussion

### 3.1. Characterization of the MXene/PEI-MWCNTs Nanocomposites

**Figure 1:**
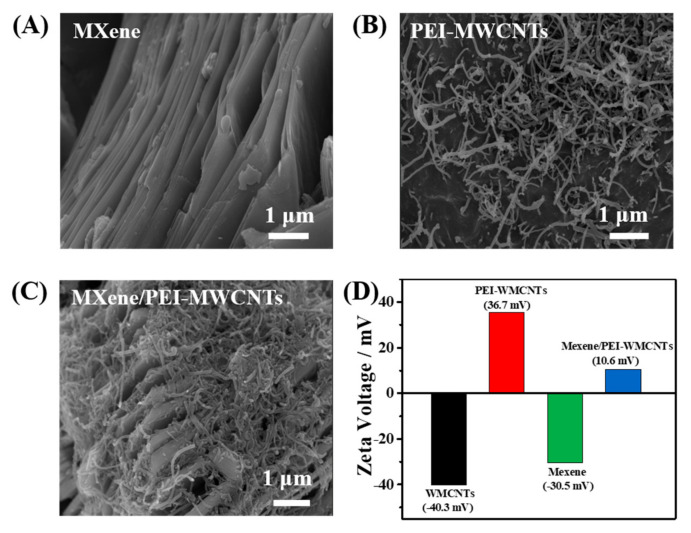
SEM images of (**A**) MXene, (**B**) PEI-MWCNTs, (**C**) MXene/PEI-MWCNTs. (**D**) Zeta potential values of different materials.

The morphology of different nanomaterials was analyzed using scanning electron microscopy (SEM). As shown in Figure 1A, MXene exhibited a two-dimensional layered structure similar to an accordion, featuring distinct interlayer spacing. The PEI-MWCNTs remained a single-layer tubular structure that is intricately intertwined (Figure 1B). Upon embedding PEI-MWCNTs into MXene, it was evident that MXene became extensively wrapped by PEI-MWCNTs, resulting in a looser multi-layer structure of MXene (Figure 1C). This indicates that upon combination with PEI-MWCNTs, it functions as an interlayer material to increase the spacing between MXene nanosheets, thereby preventing the self-aggregation of MXene. Furthermore, Zeta potentials were measured for different materials, including MWCNTs, PEI-MWCNTs, MXene, and MXene/PEI-MWCNTs, to investigate the bonding mechanism of the MXene/PEI-MWCNTs nanocomposite (Figure 1D). The distinct potentials indicate that MXene/PEI-MWCNTs is synthesized via electrostatic self-assembly of positively charged PEI-MWCNTs and negatively charged MXene.

**Figure 2:**
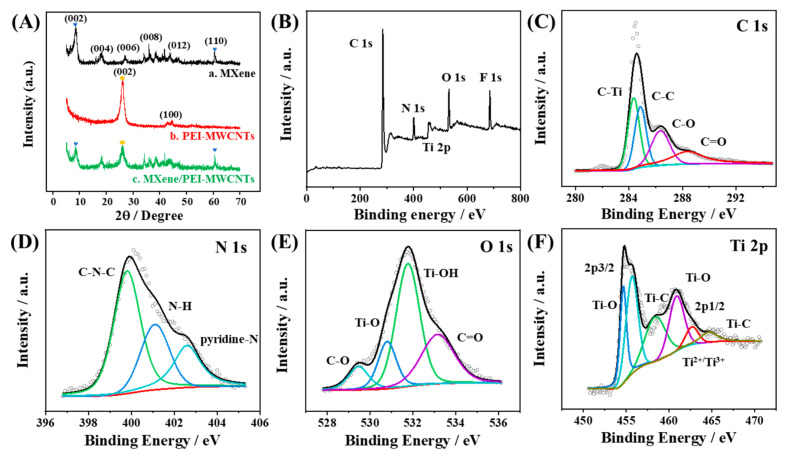
(**A**) XRD patterns of different materials. (**B**) XPS survey spectrum of MXene/PEI-MWCNTs. High-resolution XPS spectra of (**C**) C 1s, (**D**) N 1s, (**E**) O 1s, and (**F**) Ti 2p for the MXene/PEI-MWCNTs.

Furthermore, the crystal structures of MXene, PEI-MWCNTs, and MXene/PEI-MWCNTs were studied using X-ray diffraction (XRD) in Figure 2A. Representative diffraction peaks were found at the 2θ angles of 8.99°, 18.23°, 27.83°, 36.13°, 43.48°, and 60.70°, which corresponded to the (002), (004), (006), (008), (012), and (110) crystal planes of MXene (curve a) [22]. Additionally, a peak located at 25.7° was assigned to the (002) plane of the graphitic crystalline phase of PEI-MWCNTs (curve b), while the reflection peak at 43.3° corresponded to the sp^2^ hybridized diffraction structure from the (100) plane [23]. In the nanocomposite material (curve c), distinct diffraction peaks corresponding to both MXene and PEI-MWCNTs were observed. These results indicate the successful preparation of MXene/PEI-MWCNTs.

The valence states and chemical composition of the MXene/PEI-MWCNTs nanocomposite were characterized through X-ray photoelectron spectroscopy (XPS). As shown in Figure 2B, the XPS survey spectrum revealed five constituent elements: C, N, O, F, and Ti. In the high resolution XPS spectrum of the C 1s (Figure 2C), the binding energy peaks located at 284.2, 285.1, 286.2, and 288.8 eV were attributed to the C-Ti, C-C, C-O, and C=O bonds, respectively [24]. The peaks observed at 399.6, 401.2, and 402.6 eV were assigned to C-N-C, N-H, and pyridine-N bonds in N 1s spectrum [25] (Figure 2D). As shown in the O 1s spectrum (Figure 2E), the binding energy peaks at 529.4, 530.8, 531.8, and 533.1 eV were ascribed to C-O, Ti-O, Ti-OH, and C=O bonds [26]. In Figure 2F, the fitting peaks at 454.6, 455.8, and 458.3 eV for Ti 2p^3/2^ corresponded to C-Ti, Ti^2+^/Ti^3+^, and Ti-O bonds, respectively. Similarly, the peaks at 460.8, 462.4, and 464 eV for Ti 2p^1/2^ belonged to the C-Ti, Ti^2+^/Ti^3+^, and Ti-O bonds [27], respectively. These results indicate that MXene has been successfully exfoliated. The aforementioned findings further confirm the MXene/PEI-MWCNTs nanocomposite material has been successfully prepared.

### 3.2. Electrochemical Response of MIP/MXene/PEI-MWCNTs/GCE Sensor

**Figure 3:**
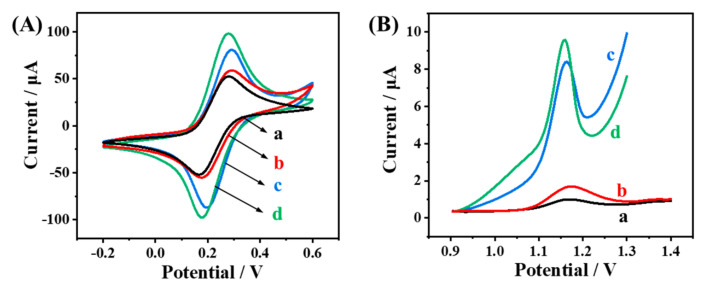
(**A**) CVs of different modified electrodes in a 0.1 M KCl solution containing 5 mM [Fe(CN)_6_]^3−/4−^ at a scan rate of 50 mV/s. (**B**) DPV responses of various modified electrodes in a 40 mM BR buffer solution (pH 6.5) containing 20 μM DU. Different modified electrodes: (a) bare GCE, (b) MXene/GCE, (c) PEI-MWCNTs/GCE, (d) MXene/PEI-MWCNTs/GCE.

The electrochemical properties of various modified electrodes were studied by means of CV technology in the 0.1 M KCl solution with 5.0 mM [Fe(CN)_6_]^3−/4−^. Figure 3A illustrated that the redox peak current of the MXene/GCE (curve b, 60.24 μA) exceeded that of the bare GCE (curve a, 57.27 μA), along with a reduced peak potential difference. This enhancement could be attributed to the large specific surface area and high conductivity of MXene [28]. Upon further modification with PEI-MWCNTs, the peak intensity increased markedly (curve c, 80.87 μA). This is because the positively charged PEI-MWCNTs lowered the negative charge density, facilitating greater access of [Fe(CN)_6_]^3−/4−^ to the GCE surface [29]. Interestingly, the current response was further significantly enhanced subsequent to the electrostatic self-assembly of MXene and PEI-MWCNTs (curve d, 98.54 μA). It is suggested that the combination of PEI-MWCNTs with MXene restrains the stacking of MXene, thereby improving the effective area and number of active sites, which effectively promotes electronic transfer [30]. Figure S1 presented the EIS results, demonstrating that the charge transfer resistance (R_ct_) of MXene/PEI-MWCNTs/GCE had the lowest value compared to other nanomaterials. This indicates that the prepared nanocomposite possesses high electrical conductivity [31].

In order to evaluate the performances of different materials in detecting DU, DPV method was employed to investigate current response of 20 µM DU on various modified electrodes. As shown in Figure 3B, a weak oxidation peak current was observed at GCE (curve a), suggesting the poor reaction kinetics of DU at bare GCE. For MXene/GCE (curve b), the oxidation peak current was gradually enhanced. This improvement maybe due to the large specific surface area of the MXene lamellar structure, which promoted the adsorption of DU at the electrode interface, thus augmenting the oxidation current of DU. Furthermore, the current signal of DU at the PEI-MWCNTs/GCE was measured to be 3.88 µA (curve c), demonstrating that the superior conductivity of PEI-MWCNTs was beneficial to amplifying the oxidation current of DU. As anticipated, the highest current response of DU is observed at the MXene/PEI-MWCNTs/GCE (curve d). This finding suggests that the combination of PEI-MWCNTs with MXene not only increases the number of active sites but also significantly enhances the electrochemical oxidation efficiency of DU at the electrode interface through synergistic interactions [32]. These results are consistent with the electrochemical characterization result, indicating that MXene/PEI-MWCNTs exhibits the optimal electrochemical performance and is highly suitable for DU detection.

### 3.3. Fabrication and Feasibility of the Ratiometric MIP-EC Sensor

**Figure 4:**
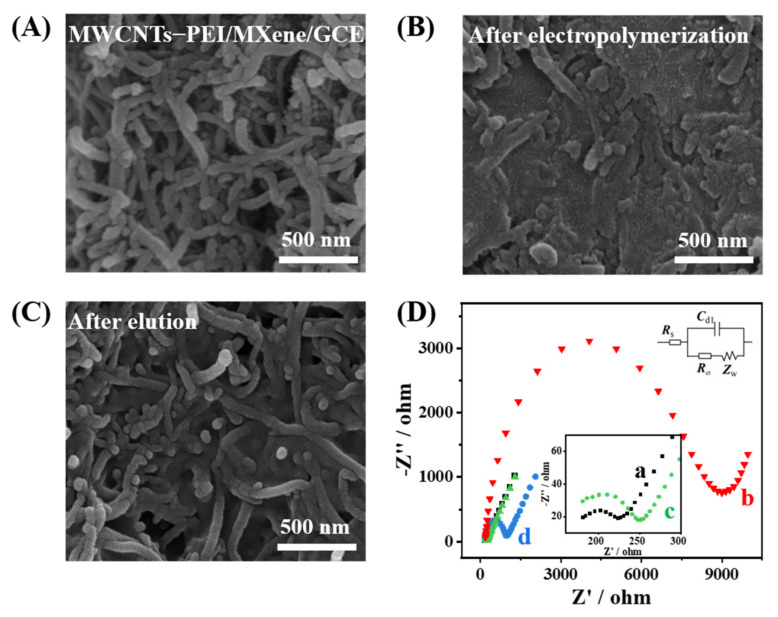
SEM images of the fabrication steps of the sensor: (**A**) MXene/PEI−MWCNTs/GCE, (**B**) after electropolymerization, (**C**) after elution. (**D**) EIS curves of the electrodes at different stages: (a) prior to electropolymerization, (b) following electropolymerization, (c) after template removal, and (d) upon 10 μM DU rebinding, the test environment in a 0.1 M KCl solution containing 5 mM [Fe(CN)_6_]^3−/4−^.

Based on the excellent conductivity and electrocatalytic activity of MXene/PEI-MWCNTs, MIP was prepared on the surface of MXene/PEI-MWCNTs/GCE via electropolymerization to develop a MIP-EC sensor. To verify the successful fabrication of the sensor, the construction process was characterized using SEM and electrochemical techniques. As shown in Figure 4A, the lamellar Mxene and tubular MWCNTs coated the surface of GCE. Following polymerization, a dense and uniform polymer film formed on the electrode surface, resulting in a relatively smooth morphology (Figure 4B). After eluting the template molecules, it was observed that the MIP/MXene/PEI-MWCNTs/GCE exhibited a loose and porous structure (Figure 4C). This structure alteration can be ascribed to the removal of DU molecules from the polymer film, resulting in the creation of specific recognition cavities [33].

Furthermore, the construction process of the sensor was further verified using the EIS method. As shown in Figure 4D, the MXene/PEI-MWCNTs exhibited good conductivity, with a R_et_ value of 45 Ω (curve a). After electropolymerization, the R_et_ value increased to 8455 Ω (curve b) owing to the poor conductivity of polymer film, which significantly impeded electron transfer. After eluting the DU molecules in an ethanol-acetic acid solution, the specific cavities that can selectively recognize DU were formed within the polymer membrane. The [Fe(CN)_6_]^3−/4−^ probe was able to access the electrode interface via these cavities, causing a decrease in R_et_ to 118 Ω (curve c). Following the rebinding process with DU, an increase in R_et_ value to 1141 Ω (curve d) was observed. This phenomenon can be ascribed to the reoccupation of the cavities by DU molecules, thereby preventing the signal probe from reaching the electrode surface. The trends of the CV signals (Figure S2) were consistent with those of the EIS. The results of the SEM and electrochemical experiments collectively confirm the successful fabrication of the MIP-EC sensor based on MXene/PEI-MWCNTs.

**Figure 5:**
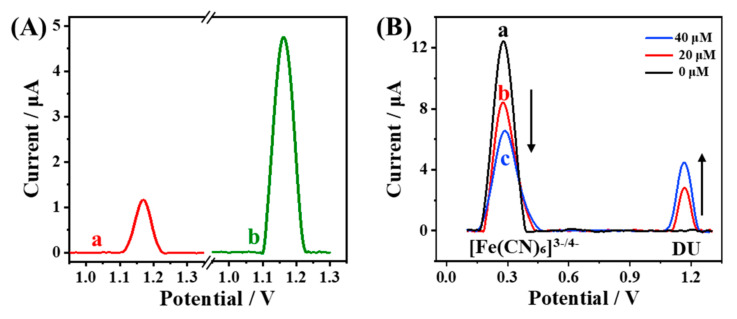
(**A**) DPVs of MIP/MXene/PEI-MWCNTs/GCE and NIP/MXene/PEI-MWCNTs/GCE towards 20 μM DU in BR buffer solution (pH 6.5). (**B**) The DPVs of the MIP/MXene/PEI-MWCNTs/GCE with diverse concentrations of DU in the BR buffer solution (pH 6.5) containing 10 μM [Fe(CN)_6_]^3−/4−^.

In order to verify the presence of cavities in MIP, DPV measurements were conducted on NIP/MXene/PEI-MWCNTs/GCE and MIP/MXene/PEI-MWCNTs/GCE after incubation with DU (Figure 5A). The peak current of the NIP-EC sensor (curve a) was notably lower than that of the MIP-EC sensor (curve b) because there were no specific imprinted sites in the NIP. Moreover, the molecular imprinting effect not only increases the electroactive surface area of the MIP but also boosts the catalytic oxidation of DU. The imprinted cavities in MIP serve as specific sites with strong affinity for target molecules, thereby facilitating selective binding. As a result, the molecular imprinting technique enables highly selective recognition and detection of DU, even in complex environments.

After introducing an inherent correction probe, the feasibility of the ratiometric MIP-EC sensor for the detection of DU was illustrated in Figure 5B. In the BR buffer solution containing [Fe(CN)_6_]^3−/4−^, where MIP/MXene/PEI-MWCNTs/GCE was not bound to DU, only the oxidation peak of [Fe(CN)_6_]^3−/4−^ could be observed, but the oxidation peak of DU was absent (curve a). Following the addition of 20 µM DU, a decrease in the peak current of [Fe(CN)_6_]^3−/4−^ was witnessed, concurrent with the emergence of an oxidation peak for DU (curve b). This phenomenon is due to the DU molecules occupying the imprinted sites in the MIP, preventing the access of [Fe(CN)_6_]^3−/4−^ to the electrode interface. When the concentration of DU was raised to 40 µM, a further reduction in the peak intensity of [Fe(CN)_6_]^3−/4−^ occurred, while the oxidation current of DU was further strengthened (curve c). The I_DU_ and I_[Fe(CN)6]_^3−/4−^ exhibited contrary trends, suggesting that the current ratio of DU to [Fe(CN)_6_]^3−/4−^ (I_DU_/I_[Fe(CN)6]_^3−/4−^) could be utilized as a signal output.

### 3.4. Analytical Performance of the Ratiometric MIP-EC Sensor

**Figure 6:**
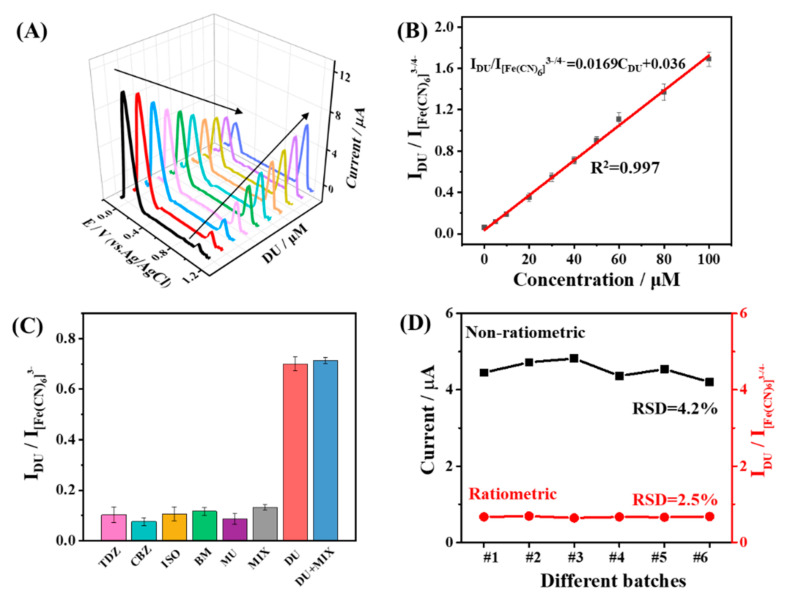
(**A**) DPV curves of the ratiometric MIP-EC sensor detect various concentrations of DU in BR solution (pH 6.5) containing 10 μM [Fe(CN)_6_]^3−/4−^. (**B**) Linear plots of the ratio responses (I_DU_/I_[Fe(CN)6]_^3−/4^) versus DU concentrations ranging from 0.1 to 100 μM. (**C**) Selectivity and the ratiometric MIP−EC sensor. (**D**) The response values of six ratiometric and non-ratiometric MIP-EC sensors.

Under the optimal experimental conditions (Figure S3), the analytical performance of the developed ratiometric MIP-EC sensor for detecting DU was investigated. With an increase in the concentration of DU, there was a gradual decrease in the [Fe(CN)_6_]^3−/4−^ signal, accompanied by an intensification of the oxidation signal of DU (Figure 6A). Consequently, the ratiometric strategy in signal on-off mode can effectively counteract the interference caused by environmental changes or instrument drift. As shown in Figure 6B, within the concentration range of 0.1–100 µM, the ratiometric signal (I_DU_/I_[Fe(CN)6]_^3−/4−^) displayed a strong linear correlation with the DU concentration. The linear regression equation was I_DU_/I_[Fe(CN)6]_^3−/4−^ = 0.0169C_DU_ + 0.036 (R^2^ = 0.997) and the limit of detection (LOD) was calculated to be 30 nM (S/N = 3). Furthermore, an examination was carried out to explore the linear relationship between the current signal of DU and its concentration under the non-ratio strategy (Figure S4). The linear regression equation obtained was I_DU_ = 0.0647C_DU_ + 1.104 (R^2^ = 0.979). This R^2^ value was notably lower than that observed with the ratiometric sensor (R^2^ = 0.997), indicating that the introduction of the ratiometric strategy can effectively minimize external interference and enhance the accuracy of DU detection. In comparison with the previously reported literature on DU detection (Table 1), the ratiometric MIP-EC sensor exhibited exceptional performance, marked by an extensive linear range and a comparatively low LOD value. It is imperative to note that the proposed sensor demonstrates superior accuracy in detecting DU owing to the innovative implementation of the ratiometric strategy.

The selectivity of the reported ratiometric MIP-EC sensor was evaluated using pesticides with structures similar to DU, including thidiazuron (TDZ), carbendazim (CBZ), isoproturon (ISO), benomyl (BM), and monuron (MU), which were chosen as interfering substances. These pesticides were tested at the same concentrations as DU (40 μM). The sensor exhibited a negligible response to these interferences and their mixtures, demonstrating a pronounced signal response specifically in the presence of DU (Figure 6C). Furthermore, the response of DU on the MIP-EC sensor was significantly higher than that on the NIP-EC sensor (Figure S5A), indicating superior specific recognition capability for DU by the MIP sensor. These findings confirm that the ratiometric MIP-EC sensor demonstrates remarkable selectivity in the detection of DU.

The reproducibility of both ratiometric and non-ratiometric MIP-EC sensors was evaluated through examining the DPV responses to 40 μM DU. Figure 6D presented the variability in the current responses obtained from six-electrode measurements. The value of I_DU_/I_[Fe(CN)6]_^3−/4−^ (RSD = 2.5%) exhibited greater reproducibility compared to the value of I_DU_ (RSD = 4.2%). This enhanced reproducibility results from the ratiometric strategy, which greatly reduces the impact of both internal and external factors, thereby improving the accuracy of DU detection. To evaluate the reusability of the ratiometric MIP-EC sensor, consecutive detection and elution cycles were performed. As shown in Figure S5B, the current signal changes of the ratiometric MIP-EC sensor (n = 3) indicated that the response retained 90.1% of its initial response after five consecutive experiments, attributed to the swelling effect of the polymer. In addition, the stability of the ratiometric MIP-EC sensor was investigated. Following a period of storage at room temperature spanning 7 days, the current response retained 92.2% of its initial response value (Figure S5C), suggesting that the fabricated sensor possesses outstanding long-term stability.

### 3.5. Real Sample Analysis by the Ratiometric MIP-EC Sensor in Soil

The practical applicability of the developed sensor was assessed in real samples and the standard addition approach was employed to quantify DU in a soil sample sourced from the Xinjiang region. In Table S1, the presence of DU was detected in the unspiked soil sample. In the spiked experiments, the recovery rates for the ratiometric MIP-EC sensor of DU ranged from 93.5% to 97.7%, with RSD values less than 4.3%. These results validated the feasibility and efficacy of the proposed ratiometric MIP-EC sensor for the detection of DU in real samples.

## 4. Conclusions

In this study, we present a novel signal on-off ratiometric MIP-EC sensor, which is designed for the selective and accurate detection of DU. The sensor is constructed with an MXene/PEI-MWCNTs nanocomposite as the substrate, offering superior electrical conductivity and a considerable surface area. The integration of the synergistic effects of the MXene/PEI-MWCNTs nanocomposite, the special recognition of the MIP, and the ratiometric strategy has resulted in the development of a promising tool for the detection of DU. The developed sensor demonstrated a wide linear range from 0.1 to 100 μM and a low LOD of 30 nM. In addition, the developed sensor was successfully utilized for the quantification of DU in cotton soil samples, with satisfactory results obtained. Compared to the single-signal MIP-EC sensor for DU detection, the prepared ratiometric MIP-EC sensing platform enhances the accuracy and stability of the sensor, thereby emphasizing its superiority over traditional MIP-EC sensors. The ratiometric MIP-EC sensor can be readily adapted for the detection of other herbicides by simply substituting the template molecules.

## Figures and Tables

**Table 1:** Comparison of the ratiometric MIP-EC sensor with other analytical methods for the detection of DU.

Detection Methods	Linear Range	LOD	Refs.
HPLC-MS	0.0858–17.16 µM	50.6 nM	[34]
GC-EC	0.0429–21.45 µM	30.0 nM	[35]
MSPD-CE-ECL	0.00858–2.15 µM	0.858 nM	[36]
ICA	0.858–42.9 nM	0.3 nM	[37]
EC	5–50 µM	360 nM	[38]
EC	0.01–764.4 µM	5.50 nM	[39]
EC	2–149 µM	2.52 nM	[7]
MIP-EC	0.1–60 µM	20.0 nM	[17]
MIP-EC	3.2–1000 µM	1.40 µM	[40]
MIP-EC	10–500 µM	43.4 µM	[41]
Ratiometric MIP-EC	0.1–100 µM	30.0 nM	This work

## Data Availability

The original contributions presented in this study are included in the article. Further inquiries can be directed to the corresponding author.

## Supplementary Materials

The following supporting information can be downloaded at: https://www.mdpi.com/article/10.3390/bios15070433/s1, Figure S1: EIS curves of various modified electrodes. Figure S2: CV responses of the construction steps of the ratiometric MIP-EC sensor. Figure S3: Optimization of experimental parameters. Figure S4: Plots of the ratio of I_DU_ to DU concentrations. Figure S5: Responses of MIP-EC and NIP-EC sensors, and stability of ratiometric MIP-EC sensor. Table S1: The results of DU in soil sample by the developed sensor (n = 3).
