# Supplementary material for: A Signal On-Off Ratiometric Molecularly Imprinted Electrochemical Sensor Based on MXene/PEI-MWCNTs Signal Amplification for the Detection of Diuron
Source: Biosensors (Basel). 2025 Jul 5;15(7):433. doi: 10.3390/bios15070433 (PMC12293771; doi:10.3390/bios15070433)
Supplement: Supplementary file 1 [file biosensors-15-00433-s001.zip › biosensors-3673418-supplementary.pdf]

Supporting Information

A Signal On-Off Ratiometric Molecularly Imprinted Electrochemical Sensor Based on MXene/PEI-MWCNTs Signal Amplification for the Detection of Diuron

Yi He [1,2], Jin Zhu [1,2], Libo Li [1,2], Tianyan You [1,2,3,*] and Xuegeng Chen [1,2]

1 Key Laboratory of Modern Agricultural Equipment and Technology, Ministry of Education, Jiangsu University, Zhenjiang 212013, China

2 School of Agricultural Engineering, Jiangsu University, Zhenjiang 212013, China

3 College of Agricultural Equipment Engineering, Henan University of Science and Technology, Luoyang 471003, China

* Correspondence: youty@ujs.edu.cn

1. Experimental section

1.1. Materials and reagents

Ethanol (C_2H_5OH), acetonitrile (C_2H_3N), potassium ferricyanide ($K_3[Fe(CN)_6]$), potassium chloride (KCl), potassium ferrocyanide ($K_4[Fe(CN)_6]$), sodium chloride (NaCl), boric acid (H_3BO_3), phosphoric acid (H_3PO_4), glacial acetic acid (HAc), hydroxide (NaOH), thidiazuron (TDZ), carbendazim (CBZ), isoproturon (ISO), benomyl (BM), monuron (MU), and sodium acetate ($C_2H_3O_2Na$) were acquired from Sinopharm Chemical Reagent Co. Ltd. (Shanghai, China). Polyethylenimide (PEI) was purchased from Sigma Aldrich (Tianjin, China). DU and o-phenylenediamine (o-PD) were bought from Aladdin Biotechnology (Shanghai, China). MWCNTs and MXene were obtained from XFNANO (Nanjing, China).

1.2 Apparatus

Scanning electron microscopy (SEM) was obtained on a HT-7800 a Apreo S field-emission scanning electron microscopy (Thermo Fisher Scientific, USA). The X-ray diffraction (XRD) pattern was carried out on Bruker D8 diffractometer (Bruker Co, German). The surface charges of different materials were measured by Zeta-sizer Nano-ZS (Malvern, UK). X-ray photoelectron spectroscopy (XPS) analysis was performed on an AXIS spectrometer (Shimadzu, Japan). Electrochemical impedance spectroscopy (EIS) was tested on 760E electrochemical workstation (Shanghai Chenhua, China).

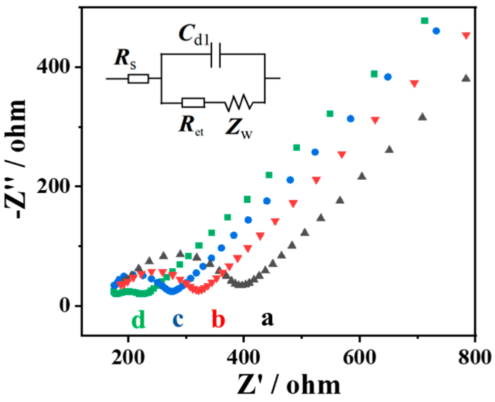

Figure S1. EIS curves of different modified electrodes in 0.1 M KCl containing 5 mM $[Fe(CN)_6]^{3-/4-}$: (a) GCE, (b) MXene/GCE, (c) PEI-MWCNTs/GCE, and (d) MXene/PEI-MWCNTs/GCE.

The impedance values of different modified electrodes were examined using EIS technology, and the results were fitted using the Randles equivalent circuit shown in the inset of Figure S1. The R_{et} values of GCE (curve a), MXene/GCE (curve b), PEI-MWCNTs/GCE (curve c), and MXene/PEI-MWCNTs/GCE (curve d) were 178 Ω, 135 Ω, 82 Ω, and 44 Ω, indicating that MXene/PEI-MWCNTs/GCE had the best conductivity and is a good substrate material for constructing electrochemical sensors.

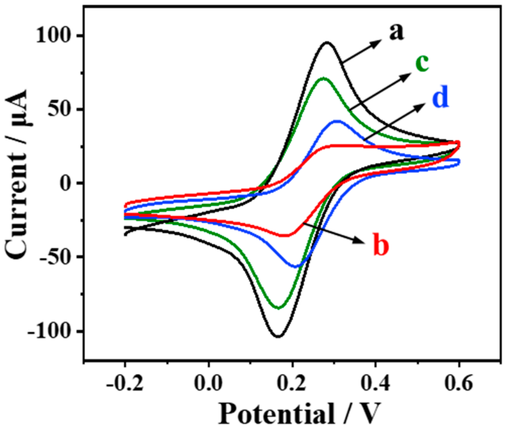

Figure S2. CV curves of the electrodes after each step: (a) before electropolymerization, (b) after electropolymerization, (c) templates removal and (d) DU rebind.

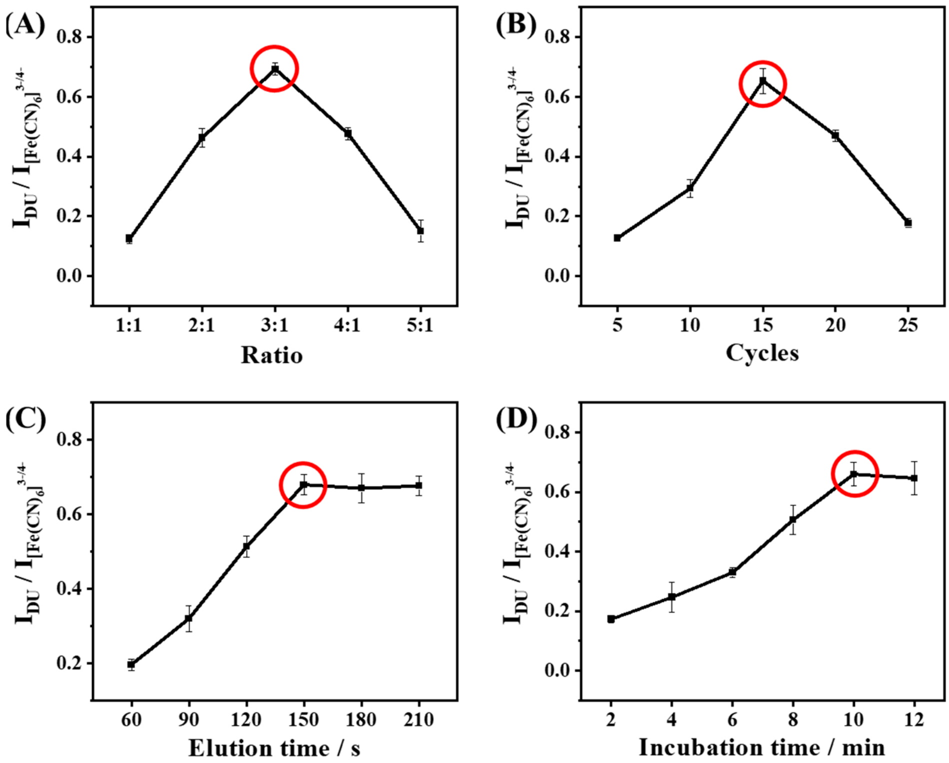

Figure S3. Optimization of experimental parameters: (A) molar ratio between monomer and template, (B) electropolymerization cycles, (C) elution time of template molecule, and (D) incubation time of DU.

In order to obtain the optimal analytical performance of the ratiometric MIP-EC sensor, the control variable method was employed to systematically optimize the experimental conditions. These conditions included the molar ratio of the functional monomer to the template molecule, the number of electro-polymerization cycles, the elution time, and the binding time. As shown in Figure S3, the optimal experimental conditions were determined to be the following: the molar ratio of functional monomer to template molecule is 3:1, the number of electropolymerization cycle is 15, the elution time is 150 s, and the binding time is 10 min.

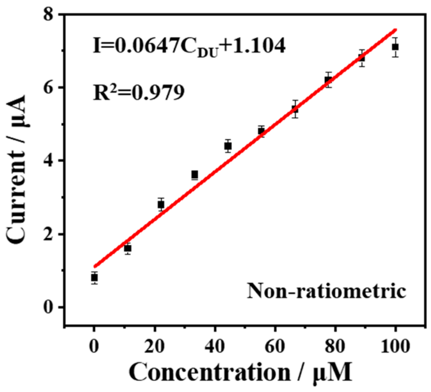

Figure S4. Plots of the ratio of I_{DU} to DU concentrations ranging from 0.1 to 100 μM.

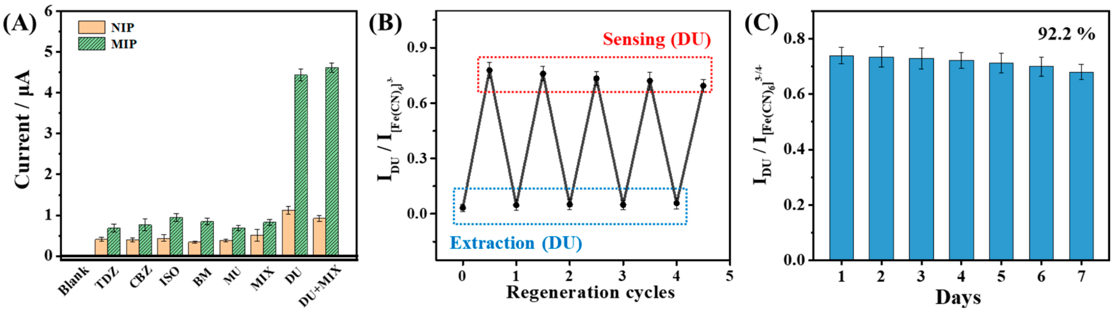

Figure S5. (**A**) Responses of MIP-EC and NIP-EC sensors in the presence of different pesticides; (**B**) Reusability and (**C**) Stability of ratiometric MIP-EC sensor.

Table S1. The results of DU in soil sample by the developed sensor (n=3)

Sample	Added (μM)	Detected (μM)	Recovery (%)	RSD (%)
Soil	0	0.1	-	4.3
	4.3	4.2	95.3	2.1
	21.5	21.1	97.7	3.1
	42.9	40.2	93.5	3.2
